# Supplementary material for: Understanding teamwork experiences of neurodivergent students: A phenomenological exploration of conflict and collaboration in engineering teams
Source: PLoS One. 2026 May 11;21(5):e0345801. doi: 10.1371/journal.pone.0345801 (PMC13160356; doi:10.1371/journal.pone.0345801)
Supplement: S1 Appendix — (DOCX) [file pone.0345801.s001.docx]

# S1 appendix: Semi-structured interview questions

## Neurodivergent status and self-identification

“In the CMS survey, you indicated that you identify as neurodivergent. Would you be comfortable sharing what condition(s) and/or trait(s) within the neurodivergence umbrella you identify with (this must include, but is not limited to ASD and/or ADHD)? Have you received a diagnosis for your condition(s) and/or traits?”

Ask about the participants’ preference for person-first (e.g., person with autism) versus identity-first (e.g., autistic person) language if applicable.

## Questions

*Probes are indicated by lettered sub-questions under each numbered question.*

### Neurodivergent identity

1. What are the strengths or benefits that stem from your neurodivergence?
   1. Consider education, work, and/or volunteer experiences.
2. What challenges stem from your neurodivergence? How do you manage/address challenges?
   1. Consider education, work, and/or volunteer experiences.

### CMS report

1. What (if anything) surprised you about your results?
   1. Do you think they are accurate?
2. Which style(s) do you rely on most and can you share an example?
   1. What thoughts or emotions came up for you?
3. What successes or challenges have you experienced in team projects as a result of your styles?
4. How do you think your neurodivergence might influence your approach to team conflict compared to neurotypical peers?

### Scenario

*Use if relevant example does not come up when prompted with “CMS Report” questions.*

Read aloud and paste into the Zoom chat for the participant to follow along and refer back to: “You have been paired with a fellow student for a student internship project. It is your first time handling this level of responsibility, and it is crucial to prove yourself to the new internship supervisor. You and your fellow intern have very different ideas about the project, and you are certain that your idea will lead to the best outcome.”

1. What details would you change or add to this scenario to make it more relatable to your experiences?
   1. What is the scenario missing based on your experiences in similar situations?
2. What thoughts or emotions might come up for you in this situation?
3. How would you envision yourself responding to conflict in this situation?
   1. Are there any strategies or actions you would consider taking?
4. Could you share any real-life situations that are similar to the scenario? How did you navigate the situation? What was the outcome or end result?

### Institutional experiences1F

1. What would help you navigate conflict in teams that you are or have been a part of?
   1. Are there any supports you wished existed?
2. What role has the university played in your experiences of team conflict?
   1. Have specific policies or expectations affected your experiences?
3. How could the university better support you in team environments and navigating team conflict?
   1. Do any ideas about changes or improvements come to mind?
4. Are there any additional thoughts or experiences related to team conflict that you would like to share?
