# Supplementary material for: Understanding teamwork experiences of neurodivergent students: A phenomenological exploration of conflict and collaboration in engineering teams
Source: PLoS One. 2026 May 11;21(5):e0345801. doi: 10.1371/journal.pone.0345801 (PMC13160356; doi:10.1371/journal.pone.0345801)
Supplement: S2 Appendix — (DOCX) [file pone.0345801.s002.docx]

# S2 appendix: Complete coding framework

| Table S2.1. Coding framework for the cognitive (sensemaking) dimension | |
| --- | --- |
| **Second Order Themes** | **First Order Concepts** |
| **Interpreting identity in team context** | Seeking understanding and validation in interactions |
|  | Discrepancy between self-image and others’ perceptions |
|  | Appreciating diversity in perspectives and experiences |
|  | Undervaluing personal contributions and skills |
|  | Developing an understanding of self and neurodivergent identity (inc. gendered experiences of neurodivergence) |
| **Exercising interpersonal sensitivity and empathy** | Perspective taking |
|  | Analyzing and understanding team members |
|  | Recognizing neurodivergent characteristics in others |
|  | Sensitivity to and awareness of others’ feelings |
|  | Sensitivity to conflict dynamics |
| **Processing internal dialogue** | Weighing the consequences (negative and positive) of self-expression |
|  | Overthinking and internal dialogue |
|  | Rigid cognitive evaluation patterns |
| **Processing emotions** | Unexpressed frustration and emotional build-up |
|  | Emotional labour of navigating neurotypical norms (inc. intersection with gender) |
|  | Self-blame, guilt, and responsibility related to contributions |
|  | Managing emotions triggered by unresolved conflict |
| **Conflict narrative** | Reframing conflict as a shared challenge |
|  | Belief that conflict is inherently harmful to relationships |
|  | Reconciling immediate vs. delayed resolution |
|  | Embracing discomfort for personal growth |

| Table S2.2. Coding framework for the behavioural (interacting) dimension | |
| --- | --- |
| **Second Order Themes** | **First Order Concepts** |
| **Approaches to participation** | Creating and pushing a vision |
|  | Encouraging inclusive and balanced participation |
|  | Prioritizing following directions as opposed to asserting ideas |
|  | Prioritizing immediate action and efficiency |
|  | All-or-nothing participation |
| **Approaches to idea exchange** | Asserting or questioning directly |
|  | Asserting or questioning with hesitance |
|  | Deferring to others’ opinions due to self-doubt |
|  | Conditional openness based on practical or project constraints |
|  | Seeking team alignment through consensus checking |
| **Conflict management strategies** | Withdrawal to avoid conflict |
|  | Monitoring to avoid conflict |
|  | Mediating and moderating to resolve conflict |
|  | Sacrificing to resolve conflict |
|  | Confronting to resolve conflict |
|  | Working in ambiguity to keep the peace |
|  | Apologizing to de-escalate conflict |
|  | Withholding contributions as a form of protest |
| **Role and responsibility distribution tendencies** | Lack of role clarity |
|  | Filling a leadership void |
|  | Situational role adaptation |
|  | Delegating for fairness and team effectiveness |
|  | Taking over tasks to ensure timely completion |
|  | Preference for individual work over collaboration |
| **Self-management and regulation** | Managing executive functioning in projects |
|  | Modelling transparency and open communication |
|  | Leveraging team support to problem solve and address personal challenges |
| **Strategies to cultivate team cohesion** | Establishing relationships to facilitate information flow |
|  | Acknowledging neurodivergent status to foster understanding |
|  | Promoting mutual respect |
