# Supplementary material for: Understanding teamwork experiences of neurodivergent students: A phenomenological exploration of conflict and collaboration in engineering teams
Source: PLoS One. 2026 May 11;21(5):e0345801. doi: 10.1371/journal.pone.0345801 (PMC13160356; doi:10.1371/journal.pone.0345801)
Supplement: Attachment — Submitted filename: PONE-D-25-53622_Response Letter_Changes Specified_V3.docx [file pone.0345801.s004.docx]

**Response to Reviewers**

Manuscript PONE-D-25-53622

Understanding Teamwork Experiences of Neurodivergent Students: A Phenomenological Exploration of Conflict and Collaboration in Engineering Teams

Thank you very much for the opportunity to revise and resubmit. We found the feedback to be extremely constructive and valuable for improving the quality of our manuscript. In this response letter, we offer responses to each comment raised. We use the following naming convention to refer to each point:

Editor, comment 1: E.1

Reviewer 1, comment 3: R1.3

| **E.0** | 05-Jan-2026  Dear xxxxx,  Thank you for submitting your manuscript to PLOS ONE. After careful consideration, we feel that it has merit but does not fully meet PLOS ONE’s publication criteria as it currently stands. Therefore, we invite you to submit a revised version of the manuscript that addresses the points raised during the review process.  ==============================  After evaluating the manuscript in light of the reviewer feedback and PLOS ONE publication criteria, I find that the study meets the journal’s standards for technical soundness, ethical conduct, and transparency of data availability. The qualitative design is appropriate for the research questions posed, and the analytic approach is sufficiently described to support the reported findings.  Changes required for acceptance:  The authors must strengthen methodological clarity regarding participant classification. Specifically, the criteria used to define the study population should be more precisely articulated, and the scope of interpretation should be aligned with those criteria. Any statements in the Results or Discussion that could be interpreted as broadly representative of clinically defined groups should be revised to reflect the actual composition of the sample. In addition, the Discussion should more clearly delineate the interpretive boundaries of the findings, particularly where broader group-based distinctions might otherwise be inferred.  Recommended changes:  To further improve clarity and reader guidance, the authors are encouraged to expand the limitations section to explicitly situate the findings within the chosen study design and sampling framework. Minor refinements to the discussion linking empirical observations to the conceptual framework would also enhance coherence, provided these links remain grounded in the data.  Resolution of reviewer advice:  There are no conflicting recommendations among the reviews. All feedback is complementary and can be addressed through clarification, reframing, and strengthened discussion, without the need for additional data collection or changes to the core methodology.  Based on the above, and consistent with PLOS ONE’s emphasis on methodological rigor and transparency rather than perceived impact, I recommend Minor Revision, with acceptance contingent upon satisfactory revision addressing the required points outlined above.  ==============================  Please submit your revised manuscript by Feb 19 2026 11:59PM. If you will need more time than this to complete your revisions, please reply to this message or contact the journal office at plosone@plos.org. When you're ready to submit your revision, log on to https://www.editorialmanager.com/pone/ and select the 'Submissions Needing Revision' folder to locate your manuscript file.  Please include the following items when submitting your revised manuscript:  A letter that responds to each point raised by the academic editor and reviewer(s). You should upload this letter as a separate file labeled 'Response to Reviewers'.  A marked-up copy of your manuscript that highlights changes made to the original version. You should upload this as a separate file labeled 'Revised Manuscript with Track Changes'.  An unmarked version of your revised paper without tracked changes. You should upload this as a separate file labeled 'Manuscript'.  If you would like to make changes to your financial disclosure, please include your updated statement in your cover letter. Guidelines for resubmitting your figure files are available below the reviewer comments at the end of this letter.  If applicable, we recommend that you deposit your laboratory protocols in protocols.io to enhance the reproducibility of your results. Protocols.io assigns your protocol its own identifier (DOI) so that it can be cited independently in the future. For instructions see: https://journals.plos.org/plosone/s/submission-guidelines#loc-laboratory-protocols. Additionally, PLOS ONE offers an option for publishing peer-reviewed Lab Protocol articles, which describe protocols hosted on protocols.io. Read more information on sharing protocols at https://plos.org/protocols?utm_medium=editorial-email&utm_source=authorletters&utm_campaign=protocols.  We look forward to receiving your revised manuscript. |
| --- | --- |
|  | **Response:** We are grateful for the opportunity to revise and resubmit our paper, and for the insightful feedback from yourself and the reviewer. The result is a much more impactful paper in our view.  Please see responses to rows E.2 and E.3 for detailed explanations of how we addressed the required and recommended changes summarized above. |
| **E.1** | Thank you for a thoughtful and timely qualitative exploration of teamwork experiences among neurodivergent engineering students. The manuscript is clearly written, methodologically coherent, and addresses an important gap in higher education research. |
|  | **Response:** We’re glad you feel our paper makes a valuable contribution. We very much appreciate your feedback, which we have incorporated into the manuscript. |
| **E.2** | Required Revisions (for acceptance)   1. Clarify Participant Characterization 2. Please provide a stronger methodological rationale for including both formally diagnosed and self-identified neurodivergent participants. 3. Clearly distinguish these groups in the Methods section and ensure that conclusions do not assume diagnostic homogeneity. 4. Reframe interpretations where necessary to reflect perceived neurodivergence rather than clinically defined categories. 5. Temper Comparative Interpretations 6. In the absence of a neurotypical comparison group, please avoid language that implies contrastive or causal claims about experiences being unique to neurodivergent students. 7. Explicitly acknowledge this limitation in the Discussion and refine conclusions accordingly. |
|  | **Response:**   1. Starting on line 219, we added a sentence to clarify participant characterization (“Students met the eligibility criteria solely based on this self-identification item; no clinical verification or diagnostic documentation was obtained.”). 2. We have added a couple of sentences to more clearly articulate the rationale for including both formally diagnosed and self-identified neurodivergent participants. Starting on line 223, we connect our inclusion criteria to the phenomenological orientation of the study. 3. Starting on line 245, we added clarifying information to distinguish between participants with and without formal diagnoses. Additionally, starting on line 817, we revised our Limitations section to clarify that findings pertain to students who self-identify as neurodivergent, which should not be interpreted as representative of diagnostically homogenous ASD or ADHD populations. 4. At the start of the Discussion section, on line 653, we specify that the patterns we identify and explain are illustrative of the sample, not all neurodivergent individuals. At the end of the Participants and Procedure section, on line 261, we clarified our use of the term “neurodivergent students,” so as not to imply that our interpretations are not specific to clinical diagnoses. 5. We tempered comparative interpretations between neurodivergent participants and neurotypical peers throughout the Discussion section. Specifically, we tweaked language to make it clear that contrasts were grounded in reported *perceptions* and that there was no neurotypical comparison group (e.g., line 667; “perceived” neurotypical norms). Furthermore, we removed sentences that implied sweeping group differences (e.g., “neurotypical team members may not always extend the same level of effort or awareness”). 6. In addition to clarifying that the patterns we identify and explain are illustrative of the sample on line 653 of the Discussion section, we rephrased sentences and terms that are causal or generalize population-level traits/differences (e.g., rephrased “…these findings suggest that neurodivergent students’ hypersensitivity to” to “Neurodivergent students’ described heightened sensitivity to…”). We also tempered or removed claims about neurodiverse teamwork, which implies a comparison group. 7. We added a limitation paragraph detailing the absence of a neurotypical comparison group starting on line 831. |
| **E.3** | Recommended Revisions (to strengthen the manuscript)   1. Strengthen the Limitations Section 2. Expand the discussion on how self-identification and lack of comparison groups may influence transferability and interpretation. 3. Briefly suggest how future studies could address these gaps (e.g., comparative or mixed-methods designs). 4. Align the Conflict Management Framework 5. Clarify whether the conflict framework is used descriptively or interpretively, and ensure that claims remain grounded in participant narratives rather than theoretical extrapolation.   Overall, these revisions primarily involve clarification and reframing, not additional data collection. Addressing them will significantly strengthen the manuscript’s methodological transparency and interpretive precision. |
|  | **Response:**   1. As outlined in E.2 responses 3 and 7, we revised our Limitations section to clarify that findings pertain to students who self-identify as neurodivergent and should not be generalized to homogenous clinical populations. We also added a paragraph detailing the absence of a neurotypical comparison group. 2. See comment above. 3. In the fourth paragraph of the Limitation section, we state, “Future researchers should employ comparative designs to examine similarities and differences across neurotypes.” 4. Not enough detail is provided for us to effectively address this comment. 5. The conflict framework is used interpretively. The results of the Conflict Management Styles assessment allowed students to reflect on how they approach conflict prior to interviews. We nested their narratives and examples they provided within the conflict framework in the Results section. Furthermore, we fine-tuned language in the Discussion section so as not to “over-extrapolate” or imply we had made a significant theoretical extension. |
| **R1.1** | This study addresses an underexplored area in higher education: the lived experience of teamwork among neurodivergent students. Despite its strengths, the study included participants who self-reported as neurodivergent, although 50% had a credible diagnosis. The study should have included all the participants with an established diagnosis.  Second, while the study integrates a conflict management framework, it does not include a neurotypical comparison group, which makes it difficult to determine which experiences are unique to neurodivergent students as compared to neurotypicals. This may limit causal or contrastive interpretation. |
|  | **Response:** We’re glad you feel our paper sheds light on an underexplored research area. We very much appreciate your feedback, which we have incorporated into the manuscript.   1. In addition to the sentences in the Participants and Procedure section explaining that including participants who self-identify as neurodivergent is a consistent practice within neurodiversity research (starting on line 220), we added a couple of sentences clarifying the methodological rationale for including both formally diagnosed and self-identified participants (see E.2 response 2 for details). 2. We added a paragraph to the Limitations section detailing the absence of a neurotypical comparison group starting on line 831 and tempered causal and comparative interpretations throughout the Discussion section (see E.2 responses 5 through 7 for details). |
